# How cell migration helps immune sentinels

**DOI:** 10.3389/fcell.2022.932472

**Published:** 2022-10-04

**Authors:** Maria–Graciela Delgado, Ana-Maria Lennon-Duménil

**Affiliations:** Spatio-Temporal Dynamics of Immune Cells Laboratory, INSERM—U932—Immunité et Cancer, Institut Curie, PSL Research University, Paris, France

**Keywords:** cell migration, actin, cytoskeleton, ameboid motility, myeloid cells, dendritic cell, tissue-resident macrophage

## Abstract

The immune system relies on the migratory capacity of its cellular components, which must be mobile in order to defend the host from invading micro-organisms or malignant cells. This applies in particular to immune sentinels from the myeloid lineage, i.e. macrophages and dendritic cells. Cell migration is already at work during mammalian early development, when myeloid cell precursors migrate from the yolk sac, an extra embryonic structure, to colonize tissues and form the pool of tissue-resident macrophages. Later, this is accompanied by a migration wave of precursors and monocytes from the bone marrow to secondary lymphoid organs and the peripheral tissues. They differentiate into DCs and monocyte-derived macrophages. During adult life, cell migration endows immune cells with the ability to patrol their environment as well as to circulate between peripheral tissues and lymphoid organs. Hence migration of immune cells is key to building an efficient defense system for an organism. In this review, we will describe how cell migratory capacity regulates the various stages in the life of myeloid cells from development to tissue patrolling, and migration to lymph nodes. We will focus on the role of the actin cytoskeletal machinery and its regulators, and how it contributes to the establishment and function of the immune system.

## 1 Introduction

The immune system orchestrates various specialized cell types and organs in complex and specialized tissue microenvironments to defend the organism. The establishment of the immune system during embryogenesis is a fascinating process in which one unique genuine hematopoietic stem cell (HSC) can support the renewal of all components of the immune system, including the lymphoid, granuloid, erythroid, and myeloid compartments (Osawa et al., 1996; Yamamoto et al., 2013; Carrelha et al., 2018).

A main cellular compartment of the immune system is constituted by cells collectively named “myeloid cells” and comprises; granulocytes, monocytes, tissue-resident macrophages, and dendritic cells (DCs). In here we find two of the best immune sentinels; the tissue-resident macrophages and DCs that sense and respond to defiant surroundings and pathogens, and in the case of tissue-resident macrophages have an essential role in tissue development, homeostasis, and remodeling (McGrath et al., 2015). The great versatility of these sentinels is in part achieved by a key feature of myeloid cells: they can efficiently migrate within and/or in between tissues. In this review, we will focus on two components of the myeloid compartment: DCs and, tissue-resident macrophages.

The amoeboid migration mode (for mechanistic details, see Box 1), is characterized by rapid single-cell crawling, which is driven by relatively fragile and transitory interactions with the substrate. This type of movement can further be divided into several cellular locomotion modes, including blebbing-based motility to gliding involving actin-polymerization. Moreover, this ameboid motility allows the cells to shift in response to changing environmental conditions or activation of distinct molecular pathways between distinct migration strategies (Lämmermann and Sixt 2009)—behavior that facilitates their entry into and movement through a variety of organs, including brain, gut, and skin. Ameboid cells hence preserve a potent capacity for recirculation between the lymphatic and blood systems as well as organs (Lämmermann and Sixt 2009).

Box 1 **Actin as a motor of Ameboid migration.**

The fundamental mechanisms underlying ameboid cell migration mainly rely on the actin cytoskeleton. Two key morphological features define a cell as “amoeboid”: (1) the cell constantly changes shape during locomotion by rapidly extending and retracting their protrusions, referred as pseudopods/“false feet,” and (2) the cell performs adhesion-independent movement based on flowing and squeezing. Such shape-driven migration leads to cell gliding or circumnavigation, generated by the polarized architecture of the actin cytoskeleton along the plasma membrane, which stiffens and contracts the cell cortex in the absence of mature focal contacts and stress fibers, avoiding degradation of extracellular matrix (ECM) barriers (as it is the case in mesenchymal migration). Importantly, while during mesenchymal migration cells exert forces that are parallel to the substrate, the one exerted by amoeboid migrating cells are rather perpendicular to the substrate (Hawkins et al., 2009).

**Formation of branched F-actin networks** requires the actin-related protein two-third (Arp2/3) complex, which starts actin filament branches on the mother actin filament existing side (Pollard 2007). Arp2/3 is activated by Wiskott–Aldrich syndrome protein (WASP) family proteins, also referred to as actin nucleation promoting factors (NPF). This family is described by its catalytic VCA domain (Miki et al., 1998). It includes: (1) WASP, was first discovered as the gene mutated in Wiskott-Aldrich syndrome patients (Derry et al., 1994); and (2) SCAR (also called WAVE), described first in *Dictyostelium* (Bear et al., 1998; Miki et al., 1998). WASP governs endocytosis and podosome formation, whereas SCAR organizes lamellipodia and pseudopodia (Campellone and Welch 2010). Recently, a number of new WASP family members was identified in the human genome (i.e WASH, WHAMM, and JMY) (Linardopoulou et al., 2007; Campellone et al., 2008; Zuchero et al., 2009). The roles of these new members are just starting to be elucidated. The small GTPases of the Rho family activates both SCAR/WAVE and WASP (Haddad et al., 2001). WASP proteins directly bind to Cdc42 (Symons et al., 1996) through their Cdc42- and Rac-interacting and binding (CRIB) domains, which favors focalized actin assembly and generate dynamic cell protrusions; lamellipodia, pseudopodia, and filopodia, allowing cell elongation and polarization. SCAR/WAVEs lacks of GTPase-binding domains, but respond to Rac as part of a complex formed by Nap1, PIR121, HSPC300 (Eden et al., 2002) and Abelson kinase (Abl) interactor (Abi), that has been shown to bind to WASP whereas PIR121 binds directly to Rac (Innocenti et al., 2005), linking WASP to SCAR/WAVE regulation. Nap1 is involved cell adhesion (Ibarra et al., 2006; Weiner et al., 2006).

**Actomyosin contractility** is the result of coordinated contraction of F-actin by Myosin II motors, leading to a retrograde flow on the plasma membrane allowing cells on solid substrates or even in liquids to progress forward. Myosin II, an hexameric protein complex that possesses two heavy chains (MHC2), two essential chains, and two regulatory light chains. On the other hand, actin, is a globular protein-producing polypeptide chains with pointed (−) and barbed (+) ends. The Myosin II regulatory light chain ATPase function produces actomyosin contractility, where MHC2 drives the translocation of actin filaments towards their (+) ends. A chain of events is tightly regulated to produce cortical tension and stiffness by the small GTPase RhoA and its effector ROCK maintaining preserved cell morphology as roundish.

The Rho family of GTPases becomes active when they are bound to GTP. A reaction that is mediated by Rho–guanine nucleotide exchange factors (Rho–GEFs). Rho–GTPase activating proteins (Rho–GAPs) catalyze instead the hydrolysis of GTP to GDP. RhoA and RhoC activate the ROCK kinases; ROCK1 and ROCK2, respectively. Upon activation, ROCK phosphorylates Myosin Phosphatase (MYPT), leading to its inactivation. In consequence, there is an increase in phosphorylated MLC2 through ROCK, leading to Myosin II activation. Rho GTPases are capable of integrating intracellular signals downstream of many receptors including; integrins, CXCR4, c-Kit, and Rho, whereas Rac, and Cdc42 (belonging to the Rho GTPase family), are initially required for actin polymerization for cells to migrate, spread and adhere (For a review see (Murrell et al., 2015).

In humans, actin-regulatory proteins deficiencies/mutations can cause primary immunodeficiency diseases (PIDs) highlighting the relevance of actin regulation in the systemic immunity (Janssen et al., 2016). For example, it has been reported that loss-of-function of actin regulators such as WASP, WIP (WASp-interacting protein), and RAC2, among others, trigger myeloid and lymphoid compartment disorder yielding to autoimmune disease, PIDs and cancer (see (Burns et al., 2017) for review).

Certain migrating blood cells, i.e. lymphocytes and some myeloid cells (DCs and tissue-resident macrophages) exhibit an amoeboid motility (von Andrian and Mempel 2003; Lämmermann et al., 2008; Jacobelli et al., 2010; Collin and Bigley 2018). This is constrained to cells that migrate towards porous environments, that do not need an opening of junctions for cells to progress or proteolysis of the extracellular matrix (ECM) (Wolf et al., 2003). The case of macrophages is more complex. Macrophages exhibit two types of motility in a 3D environment: amoeboid and mesenchymal (see Box 2). They perform amoeboid migration when migrating through porous matrices, for example in fibrillar collagen I matrix, whereas they execute mesenchymal migration–comprising matrix proteolysis and generation of false-feet (referred to as podosomes) in compact matrices like Matrigel (Friedl and Weigelin 2008; Van Goethem et al., 2010). In this review, we will focus mainly on ameboid myeloid cells (DCs and macrophages), but we will keep in mind that macrophages can switch to mesenchymal migration according to the composition of their surroundings. The mechanism of invasion and penetration of extracellular or cellular barriers, such as endothelial, epithelial, or basement membranes involve other cellular principles, which are discussed elsewhere (Ley et al., 2007; Rowe and Weiss 2008).

Box 2 **Mesenchymal migration.**

Mesenchymal cell migration is a motility mode that involves adhesion- mediated by cell actin-rich protrusions and often accompanied by ECM modification. Adhesion of protrusions formed at the cell leading edge is followed by retraction of the contractile cell rear, which is accompanied by disassembly of adhesions and rear detachment. Integrins mediate adhesion to the extracellular matrix (ECM). They correspond to non-covalently linked α/β heterodimers constituting a family of cell surface adhesion receptors. It has been described 16 α and 8 β subunits, sub-divided into families sharing β subunits, originating 22 different heterodimers. Some integrins mediate cell–cell adhesion; like α4 and β2 subunit–containing integrins on leukocytes. The basal membrane surrounding lymphatic vessels contains an intricate mixture of ECM proteins, including proteoglycans, type IV collagen, laminin, as well as fibronectin. Proteolytic extracellular matrix remodeling is performed by Matrix metalloproteinases (MMPs), (for a review see (Bear and Haugh 2014)).

During mammalian embryonic development, myeloid cell compartment is shaped after three consecutive waves of hematopoiesis (details in Box 3). A myeloid progenitor rising from the Yolk sac generate microglia in the brain during the most primitive wave of hematopoiesis. Then, during the second wave, a multipotent erythro-myeloid progenitor originates the tissue-resident macrophages: Langerhans cells in the skin, Kupffer cells in the liver and alveolar macrophages in the lung. Later, at embryonic day 10.5 (E10.5) in mice a third wave of fetal liver hematopoiesis occurs; fetal liver-derived monocytes differentiate into tissue macrophages; furthermore, they also contribute to the Langerhans cell pool in the epidermis, the lamina propria macrophages in the gut (Yosef and Regev 2016), and seed the lung just before birth, this later will constitute the long-lived alveolar macrophages (AM) pool. The myeloid compartment is highly dynamic and can adapt its behavior to respond to surrounding challenges (during homeostasis or inflammatory response), modifying its components differentiation, tissue infiltration, and activation status (Yosef and Regev 2016).

Box 3 **Fetal hematopoietic development.**

This is achieved in three consecutive waves of hematopoiesis that lead to the establishment of the hematopoietic system.

1) The first wave occurs during mice embryonic day 6.5 (E6.5) and last until E8.5, and is referred to as “primitive hematopoiesis.” Myeloid progenitors appear in the primitive ectoderm at the Yolk sac and originates primitive (fetal-type) macrophage, megakaryocyte and erythroid progenitors. These primitive macrophage progenitors eventually differentiate into brain microglia. Between E 7.0 and E 7.5, so-called blood islands appear in the visceral Yolk sac. Later, from E8.0 until E 9.0, blood island’s outer layer cells of the acquire a spindle shape and give rise to the endothelial cells.
2) The second wave is characterized by the development of multipotent erythro-myeloid progenitors in the Yolk sac, that emerge at approximately E8.5 producing definitive (adult-type) erythrocytes and erythro-myeloid progenitors that will differentiate into tissue-resident macrophages, that colonize and seed diverse tissue niches (except the brain) and exhibit tissue-specific functions.
3) The third proposed wave starts by the emergence of multipotent progenitors around E9 in both; Yolk sac and intra-embryonic tissues; including the para-aortic splanchnopleura and aorta-gonad-mesonephros. Some of these progenitors appear as predisposed toward a lymphoid fate (i.e. lymphoid-primed multipotent progenitors). These progenitors migrate and seed the fetal liver, and represent a long-lived pool maintain throughout adulthood.

In contrast to the first wave of primitive hematopoiesis, the second and third waves emanate from specialized endothelial cells, called hemogenic endothelial cells, which are present in both YS and para-aortic splanchnopleura and aorta-gonad-mesonephros, and only during a restricted period of time (E8.5–11.5) display hematopoietic activities. Long-term adult-repopulating HSCs precursors (known as pre-HSCs) are distinguishable in the intra-embryonic aorta-gonad-mesonephros at the end of the third wave (around E10). The last hematopoietic cells to appear are the long-term HSC at around E11, these ones are capable to self-renew and long-term repopulating all hematopoietic lineages in engraftment assays. Most probably these cells arise from hemogenic endothelial cells- differentiated from pre-HSCs in the aorta-gonad-mesonephros region followed by the Yolk sac and placenta. The last wave of fetal hematopoiesis, in which Long term-HSC development begins in fetal life (mid-gestation) continues generating all hematopoietic lineages during adulthood (for a review see (Epelman et al., 2014)).

We will summarize the initial hematopoietic events occurring during the embryogenesis resulting in the establishment of the myeloid cell compartment. We will see that few is known about the movement nature during the establishment of the compartment. This lack of knowledge is mostly due to three factors: the difficulty to perform *in vivo* studies; the fact that key events occur at minuscule time scales; and the observation that isolated HSCs *in vitro* do not behave identically to *in vivo* cells, and seem to require their surrounding environmental cues to guide their developmental path. This is prominently exemplified by the failures of cell engraftment studies in the context of immunodeficiencies (Epah and Schäfer 2021).

We will analyze the evidence supporting the myeloid cells migration’s molecular mechanisms and how the extracellular microenvironment regulates this migration process. Finally, we will address why the migration of myeloid cells is key for maintaining the homeostasis of tissues.

## 2 Formation of the myeloid cell compartment

### 2.1 Migration of fetal hematopoietic precursors

The ability of the immune system to build organized myeloid compartments throughout the entire organism critically depends on spatiotemporal coordination of myeloid cell migration and positioning in specific localizations known as “niches” (Petrie Aronin et al., 2017). In both mice and humans, during embryonic development, cells proliferate, differentiate and assemble themselves into layers, referred to as “germ layers,” each of which give rise to specific tissue or organs.

In the gastrulating phase of embryonic development, a one-dimensional cell layer (blastula), rearranges into a multi-dimensional structure called the gastrula, formed by three tissue layers; endoderm, mesoderm, and ectoderm. During the organogenesis, each specific primitive systems will be originated from each of these layers (Mikawa et al., 2004). The endoderm and mesoderm are at the edge of our focus, since they will give rise to lymphatic system (endoderm), the bones, the muscles, and circulatory system of the embryo (mesoderm). Additionally, the mesoderm counts for the establishment of extra-embryonic structures, that protect and nourish the embryo; the Yolk sac, the umbilical cord, and the placenta (Saykali et al., 2019).

The three crucial components of the immune system; Macrophage, lymphatic, and hematopoietic systems develop independently from each other as reported by exhaustive phylogeny and ontogeny studies (Anastassova-Kristeva 2003). The earliest chronologically is the macrophage system, which arises as ameboid cells in the embryonic mesenchymal compartment. As immune sentinels, these cells are described by their capacity to distinguish “self” from “non-self” and ingest external elements *via* phagocytosis (Metchnikoff 2012). At E7 in mice, primitive macrophages emerge (Palis et al., 1999), even before the blood circulation onset (Ghosn et al., 2019). The ultimate component to emerge is the hematopoietic system, which derives from the splanchnic mesoderm of the Yolk sac (Pardanaud et al., 1989). This “hematogenic” tissue contains a common precursor cell, termed “hemangioblast” (Sabin, 1920), which gives rise to endothelial cells. These endothelial cells in turn generate sinusoidal endothelium and HSCs–the bone marrow stem cells that will generate myeloid cells, erythrocytes, and megakaryocytes–in an anatomic area referred to as “blood islands” (Moore and Metcalf 1970; Palis and Yoder 2001). Both cell types persist committed to the outer wall of the vitelline vessels, representing a competent mechanism for infiltrating the hematogenic tissue into the developing embryo (Anastassova-Kristeva 2003).

### 2.2 Migration of definitive hematopoietic precursors to fetal liver and bone marrow

**FIGURE 1:**
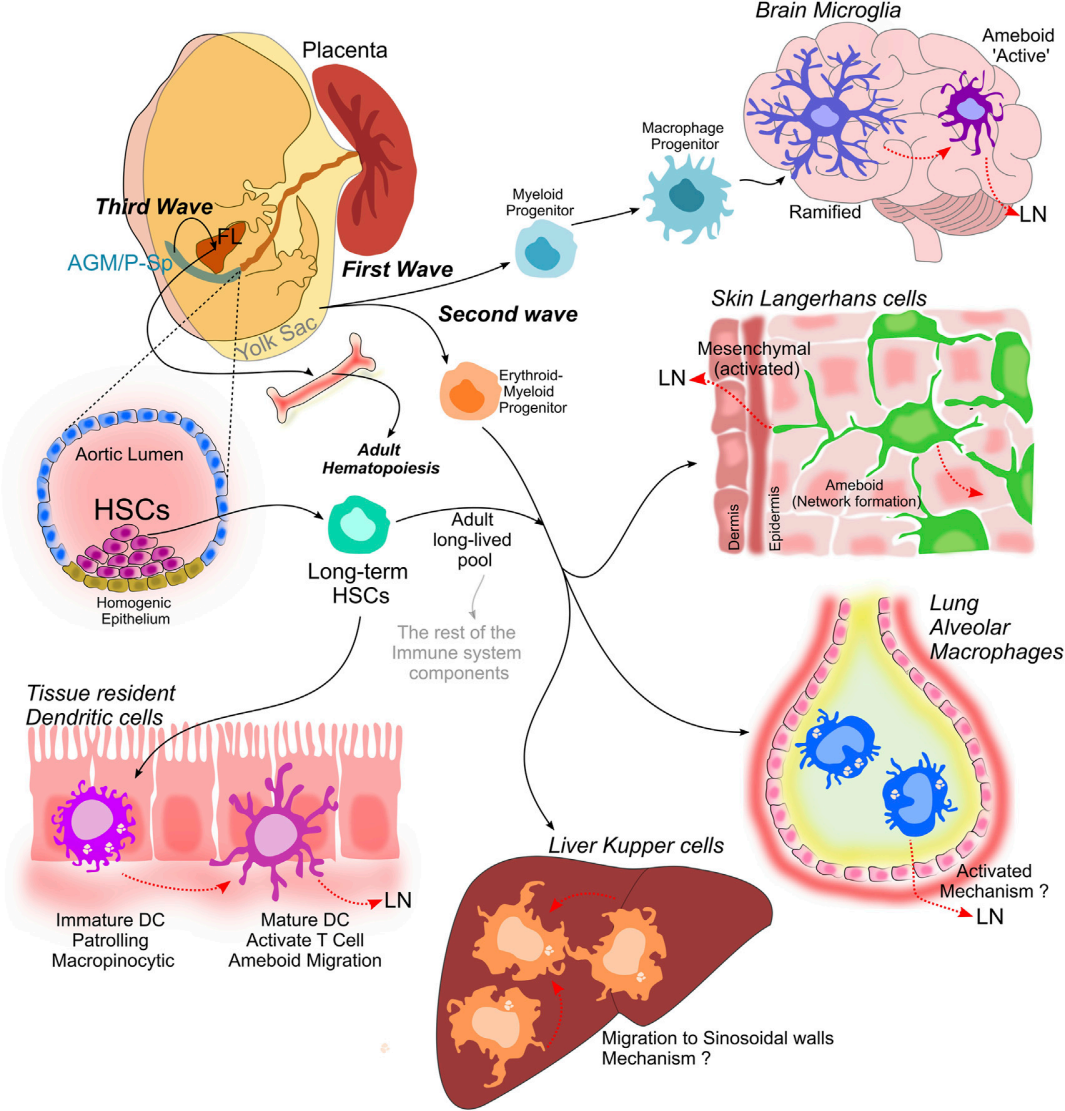
Myeloid cell compartment establishment. Is achieved during the three consecutive waves of hematopoiesis that lead to the establishment of the hematopoietic system. Myeloid progenitors develop from the Yolk sac and originate macrophage progenitors that later differentiate into microglia in the brain, that transit from a ramified state to an ameboid phenotype when activated. The second wave also occurs in the Yolk sac and it is characterized by the development of multipotent erythro-myeloid progenitors, which give rise to tissue-resident macrophages: Langerhans cells in the skin, alveolar macrophages in the lung and Kupffer cells in the liver. A third proposed wave in which multipotent progenitors emerge in both the Yolk sac and intra-embryonic tissues, including the para-aortic splanchnopleura (P-Sp) and aorta- gonad-mesonephros (AGM), give rise to long term-HSCs that migrate and colonize the fetal liver (FL), and represent a long-lived pool that will last throughout adulthood. These long term-HSCs are capable to self-renew and long-term repopulate all hematopoietic lineages during adulthood, and give rise to dendritic cells, that are present in all tissues.

Hematopoietic progenitors from all waves of fetal hematopoiesis (details in Box 3, illustrated in Figure 1) circulate through the blood vessels and seed the fetal liver, spleen and bone marrow hence supporting homeostasis (See Box 4). Hematopoiesis elapse within the different microenvironments that HSCs face during fetal and adult development, concomitantly to temporally and spatially delivered signals; growth factors, cytokines, and chemokines, together with cell-to-cell–mediated signaling (Ciriza et al., 2013). At E10, definitive HSCs arise from the aorta-gonad-mesonephros (AGM) and generate all immune lineages (Moore and Owen 1965; Moore and Metcalf 1970). At this stage in development, there is no hematopoietic cell diversity and restricted Yolk sac progenitors originate only red blood cells and macrophages, which are the single ‘white blood cell’ existing so far.

Box 4 **Hematopoietic progenitors seeding fetal liver, spleen and bone marrow.**

Early hematopoietic progenitors seed and colonize the fetal liver, where they proliferate and mature regardless of their site of origin. Later on, these hematopoietic progenitors seed the fetal spleen and bone marrow. In the bone marrow they generate a small pool stem cells from which hematopoietic cells will continually differentiate during the entire life of an animal (Godin and Cumano 2005).

**Fetal Liver:** From E11 onwards, HSCs migrate and rapidly expand into the hematopoietic organ rudiments (i.e. fetal liver, spleen and bone marrow) (Ikuta and Weissman 1992; Morrison et al., 1995; Ema and Nakauchi 2000). Around E11.5-E12 (Kumaravelu et al., 2002; Christensen et al., 2004), Long term-HSCs, derived from Yolk sac- and para-aortic splanchnopleura and aorta-gonad-mesonephros-derived progenitors, migrate to the mouse fetal liver (and placenta). Once established in the fetal liver, the long term-HSCs proliferates and originates a hierarchical hematopoiesis, leading to the generation of the vast majority of immune cells (adult-type), including circulating follicular B and T lymphocytes, granulocytes, and monocytes.

**Spleen:** Fetal spleen begins to be colonize by hematopoietic cells at E12.5 (Delassus and Cumano 1996; Godin et al., 1999). However, active spleen seeding is detected by E15.5, with daily incremental hematopoietic activity until E17.5 (Christensen et al., 2004). Unlike fetal liver cells, hematopoietic cells within the spleen do not significantly proliferate (Godin et al., 1999). At days 2 and 14–15 post birth (P), two peaks in the spleen of the ‘colony-forming progenitor cells’ (CFCs) can be distinguished (Wolber et al., 2002). The fetal spleen is seeded initially by progenitors unable to sustain myelopoiesis, suggesting that long term -HSC seeding and maintenance within this organ elapsed with a parallel mechanism (Christensen et al., 2004), in which a more differentiated and/or distinct hematopoietic progenitor, shifts the first progenitors seeding fetal spleen (Godin et al., 1999). However, how HSCs migrate towards the fetal spleen and expand to a specific hematopoietic linage remains to be elucidated.

**Bone Marrow:** Vascularization of the fetal bone marrow allows blood circulation towards the bones and enables HSCs and hematopoietic progenitors (HSCPs) to seed the marrow cavity (Aguila and Rowe 2005). Although clonogenic progenitor activity is already detectable by E15.5, the first functional long term -HSCs in the fetal bone marrow can be found only at E17.5 (Christensen et al., 2004; Gekas et al., 2005). It is thought seeded elapses concomitantly to the bone development and it is achieved by few HSCs seeding the limb bud just before the bone establishment (Kumaravelu et al., 2002). Hematopoiesis sustained by long term -HSC is established at about E18 in the bone marrow (Christensen et al., 2004). and supports blood and immune system regeneration during the entire organism life (Sawai et al., 2016; Säwen et al., 2018).

Blood cell migration is drastically affected by experimentally induced or naturally occurring mutations in genes encoding cell-adhesion receptors (Anderson and Springer 1987; Labow et al., 1994; Tedder et al., 1995b; Subramaniam et al., 1995; Yang et al., 1995). It has been shown that absence of β-integrins compromised migration but not HSC differentiation (Hirsch et al., 1996), supporting the notion that adhesive interactions facilitated by β1 subfamily of integrins (Hynes 1992) are essential to control blood cell precursors migration (Giancotti et al., 1986; Patel and Lodish 1986; Williams et al., 1991; Ruiz et al., 1995). β1-integrin subfamily members are required for migration, including both binding to the basement membrane as well as adhesion to endothelial cells (Springer 1994). Indeed, lack of β1-integrin does not affect blood cell differentiation, but inhibits arrival, so-called “homing,” to the fetal liver (Hirsch et al., 1996). In sum, these data suggest that HSC use integrins for migration, excluding them as ameboid-migrating cells, which is surprising because once they find their niche, tissue-resident macrophages derived from HSC behave as ameboid cells, and their entire function relies on this feature.

Research on the signaling controlling cell migration, using dual-chamber based migration analyses, revealed that chemotactic behavior is different for fetal and adult HSCs. Both migrate in response to chemokine stromal cell-derived factor-1α named CXCL12, but additionally fetal HSCs significantly respond to the cytokine SLF. Effectively, all fetal HSCs in this assay become migratory in response to CXCL12 and SLF combination, highlighting synergic effects in the migration of fetal HSCs in response to two SLF and CXCL12 chemoattracts (Christensen et al., 2004). A further implication of this finding is that HSCs, which circulate at low levels constitutively, are not required in large fluxes entering the fetal blood circulation, when seeding the fetal hematopoietic tissue, during the gestational period from E12 to E17. Likewise, HSCs number noticeably augment in the fetal liver (from 1,000 at E12.5 to more than 5,000 at E14.5 approximately) (Ikuta and Weissman 1992; Morrison et al., 1995; Christensen et al., 2004). However, this increase is only transient; by E15.5–16.5, the number of fetal liver HSCs drops as they migrate to seed the spleen and the bone marrow (Morrison et al., 1995; Ema and Nakauchi 2000; Christensen et al., 2004). Hematopoietic niches in the spleen and bone marrow, are seeded progressively by HSCs meanwhile they develop and support HSC self-renewal. Genetic deficiency of CXCL12 or its receptor; CXCR4 in mice, leads to normal fetal liver hematopoiesis but animals are unable to establish bone marrow hematopoiesis (Nagasawa et al., 1996; Zou et al., 1998; Ara et al., 2003).

### 2.3 Colonization of bone marrow by hematopoietic stem cells

Toward the end of prenatal life, second trimester in humans approximately, the liver is “invested” with HSCs and remains the central hematopoiesis organ. At that stage, HSCs find their adulthood niche after translocating through the peripheral circulation, guided by signals from cytokines and chemokine receptors, to the bone cavities, leading to the establishment of the so-called bone marrow (Christensen et al., 2004). During adult life, HSCs migrate continually between blood and bone marrow (Méndez-Ferrer et al., 2008), passing transitory intervals in the circulation. It has been estimated that when peripheral circulation is joined in mice, more than 99% of the HSC pass less than 6 s in the circulation (Wright et al., 2001). Nevertheless, HSC migration from fetal liver to fetal bone marrow is a process that remains to be fully understood.

As we described in the previous section, the chemokine CXCL12 and its receptor CXCR4 are key regulators of fetal liver HSC migration but also during adult life (Wright et al., 2002). The CXCL12 /CXCR4 pathway regulates the expression of extracellular matrix assembly and degradation enzymes, including; gelatinases A (MMP-2) and/or B (MMP-9). In bone marrow, MMP2 upregulated expression in hematopoietic progenitor cells (CD34^+^ progenitors) is achieved by facilitating factors of cell mobilization into the circulation, such as the granulocyte-colony stimulating factor (G-CSF) and stem cell factor (SCF, also known as c-Kit ligand) (Janowska-Wieczorek et al., 1999). Remarkably, cleavage of CXCL12 at positions 4–5 is catalyzed by MMP2 (McQuibban et al., 2001). The CXCL12 remaining fragment (position 5–67) is unable to produce the chemo-attractive response in human CD34^+^ progenitors since is unable to bind to the receptor CXCR4 (McQuibban et al., 2001). The cleavage of CXCL12 resulted from G-CSF–induced MMP2 catalysis might represent a mechanism of controlling the release of human CD34^+^ progenitors into circulation (Ciriza et al., 2013). Also, since circulating human CD34^+^ cells have been reported to express MMP2 and MMP9, this suggests that their migration occurs is a non-ameboid fashion, and that they migrate toward ECM modifications, whereas bone marrow CD34^+^ cells do not, which is reflected by their ability to migrate into Matrigel-based matrices (Murphy and Knäuper 1997; Janowska-Wieczorek et al., 1999).

Additionally, it has been shown that bone marrow stromal cell-derived CXCL12 stimulates CD34^+^ cells migration and CD34^+^ cells after reinfusion into peripheral veins are able to migrate to the bone marrow. A study comparing spontaneous and CXCL12 -induced migration across Transwell filters of CD34^+^ cells from bone marrow, peripheral blood, and cord blood (Voermans et al., 1999) elucidated the migration determinants. Cord blood CD34^+^ cells are highly migratory compared with the other two cell populations. Also, higher CXCL12 induced migration was observed in bone marrow derived CD34^+^ cells suggesting different sensitivity to CXCL12. Accordingly, expression of the CXCR4 in CD34^+^ cells from peripheral blood was found lower compared with bone marrow and cord blood CD34^+^ cells. Nevertheless, no changes in sensitivity to CXCL12 were determined after measuring migration towards different concentrations of CXCL12 for bone marrow and cord blood derived CD34^+^ cells, in agreement with CXCR4 receptor expression. On the other hand, when ECM protein fibronectin was used to coated filters, an increase CXCL12—induced migration was observed for peripheral blood and bone marrow CD34^+^ cells (2.5 and 1.5 times increase, respectively) and was blocked after treatment with antibodies against β1-integrins. All together these observations suggest that increased migration of cord blood derived CD34^+^ cells may favor homing to the bone marrow (Voermans et al., 1999).

The bone is formed by an exceptional mesenchymal cell type called osteoblast, together with characteristic extracellular matrix glycoproteins and an exceptionally high density of calcium salts. Inside this rigid architecture is found the bone marrow, the well-known blood cell production site, a combination of differentiating hematopoietic cells in areas circumscribed by trabecular bone, endothelium-lined sinuses, blood vessels, and adipocyte tissue (Adams and Scadden 2006). While the role of bone in marrow physiology remains unclear, the HSC population has been characterized by ‘immuno-phenotype’, nevertheless, its specific location within the bone marrow has been refractory to analysis, because this mainly fluid tissue exists inside the stiffest tissue: the bone (Adams and Scadden 2006). A unique HSCs- regulatory niche is achieved in this complex microenvironment, as shown by *in vivo* studies, there is an association between hematopoiesis and osteogenesis. It has been shown that Cbfa1/Runx2, a transcription factor important for osteoblast progression and chondrocyte development, deficient mice embryo have normal hematopoietic development until E17.5, but at E18.5 both liver and the spleen display excessive extramedullary hematopoiesis (Deguchi et al., 1999). Also, it has been shown that collagen X, an important molecule for bone formation leads to a similar perinatally phenotype in mice (Gress and Jacenko 2000; Jacenko et al., 2001, 2002).

HSC migration is also affected by calcium gradients, it has been shown that the concentration of Ca^+2^ in endosteal area of the bone marrow increases together with bone mineralization. It has been shown that CASR, a seven membrane–spanning G-protein coupled calcium-sensing receptor, is expressed by HSCs allowing them to respond to extracellular calcium concentrations. Lack of CASR in HSCs leads to impairment to collagen I adhesion, an ECM protein secreted by osteoblasts, resulting in inability to arrive in the endosteal bone marrow niche. CASR -deficient mice, display bone marrow hypo-cellularity and extramedullary hematopoiesis at neonatal stage, suggesting that CASR is required for proper HSCs migration and homing to the bone marrow niche (Adams and Scadden 2006). Nevertheless, it is not required during embryonic development, as CASR-deficient embryos do not display changes in the number of HSC in fetal liver.

Studies performed in bone marrow -derived HSCs show them in motion. Moreover, *ex vivo* imaging studies have suggested that they display dynamic membrane protrusions associated with a rapid and directed motility (Frimberger et al., 2001). Mimicking the homing event has been achieved by co-culturing HSCs and stromal cells, this *in vitro* homing assay has shown that individually HSCs are highly migratory, displaying motile and long pseudopodia-like membrane extensions. Also, it was observed that the cell movement is rather directional in response to CXCL12 and steel factor. Interestingly, in response to extreme pH (8.5–9.5), an increase of size and number of pseudopodia-like membrane extensions was observed (Allena 2013). Mature cells of the immune system may be considered to possess motility as a necessary adaptation for fast and efficient surveillance in the context of host defense. However, an explanation for these phenomena in stem cells is less teleologically intuitive. Do stem cells perform some kind of monitoring function? Such functions are not obvious in general, but bone marrow –derived cells do integrate into neovascular tissue, suggesting that some participation in repair is likely (Udani et al., 2005).

So far, we have discussed the establishment of a circuit from Yolk sac to fetal liver and from fetal liver to fetal bone marrow, that drives the establishment of the myeloid compartment. The bone marrow becomes colonized just before birth in mice and during the second trimester in humans, leading to the production of a HSCs minor pool in which hematopoiesis relies on during adult life (Godin and Cumano 2005). This implies that HSCs are capable of self-renewal and multilineage differentiation within the bone marrow niche (Adams and Scadden 2006; Arai and Suda 2007). HSC functions are orchestrated by both, intrinsic and extrinsic factors at a fine-tuning scale (Scadden 2006; Kiel and Morrison 2008). For example, hematopoietic lineages express both Rac1 and Rac2, but at the progenitor level (HSPCs) they perform distinct functions (Matsuoka et al., 2001). Rac1-deficient HSPCs show impaired hematopoietic repopulation due to reduced position within the tissue rather than defective long-term re-population potential when engraft (Gu et al., 2003; Cancelas et al., 2005, 2006). Whereas, Rac2-deficient HSPCs are able to engraft but repopulation is impaired, suggesting defective adhesion to and weakened retaining within the bone marrow niche (Gu et al., 2003; Cancelas et al., 2005, 2006). Regarding the Rac downstream effectors involvement in the HSCs engraftment process, it has been reported that knock-down (KD) of WAVE2 in HSCs displays modest early bone marrow repopulation similar to Rac1-deficient HSC phenotype, suggesting that after engraftment, WAVE2 might be the specific effector of Rac1 in HSCs, during bone marrow early repopulation (Ogaeri et al., 2009).

On the other hand, Cdc42; another Rho GTPase family member, has been also been involved as a downstream effector. Cdc42-deficient HSCs are unable to migrate across a Transwell, or across an endothelial monolayer, following a CXCL12 gradient (Yang et al., 2007), leading to fail at bone marrow homing, retention, and long-term repopulation. Also, Cdc42 was shown as a positive regulator of HSC quiescence status, suggesting Cdc42 is critical for functional and physical interaction between HSCs and bone marrow niche. This has been shown to occur through N- WASp and WASp pathways that lead to filopodium formation (Takenawa and Suetsugu 2007), isolated Cdc42-deficient HSPCs display abnormal filamentous actin (F-actin) reorganization after CXCL12 stimulation. Taken together, these data show that Cdc42 is required for proper HSC actin reorganization, directional migration and progenitor cell adhesion supporting HSC retention and homing at the bone marrow niche (Yang et al., 2007). Whether these phenotypes reflect Cdc42 homeostatic role in normal HSCs is unknown, because Cdc42-deficient mice are not viable.

## 3 Homeostasis of the adult myeloid compartment

We will focus now on the evidence supporting the migration of adult myeloid cells in an homeostatic context, with a focus on DC and macrophages (Hashimoto et al., 2013). Macrophages establish a wide family of professional phagocytic cells that reside within all tissues and includes brain microglia, epidermal Langerhans cells, lung alveolar macrophages, and liver Kupffer cells. This compartment plays an important role during tissue development and homeostasis (Ginhoux et al., 2016; Mass 2018) besides defense against invading pathogens.

Resident macrophages are prenatally established in virtually all tissues from embryonic precursors (see Box 3) and they are self-renewably maintained independently of monocytes (Merad et al., 2002; Ginhoux et al., 2010; Hoeffel et al., 2012, 2015; Schulz et al., 2012; Guilliams et al., 2013; Hashimoto et al., 2013; Yona et al., 2013; Gomez Perdiguero et al., 2015; Sheng et al., 2015).

Importantly, Macrophages orchestrate almost all main diseases, i.e. inflammatory (rheumatoid arthritis) & chronic neurodegenerative diseases, infection, sepsis, and also cancer (for a review, please check (Ardura et al., 2019). Likewise, DC migration dysregulation leading to anomalous positioning or activation of DCs, results in mistuned immune responses and even immune diseases, including; allergies, autoimmune diseases, and tumors (for a review, please check (Liu et al., 2021)).

### 3.1 The dendritic cell sub-compartment

DCs are the antigen-presenting cells, defined by their distinct morphology (Steinman and Cohn 1973) are found at the interface of innate and adaptive immune responses. They have been classified primarily by their ontogeny, and secondarily by their phenotype, function and location (Guilliams et al., 2014). In this section we will described the characteristic phenotype of DCs; immature and mature and then we will explore the diversity of DC populations, considering that four major DC subsets have been described and classified.

During myelopoiesis, a myeloid precursor can choose between two routes of differentiation that will originate; megakaryocytes, erythrocytes or granulocytes, or will become a macrophage/DC precursor (MDP) that will be capable of differentiating into plasmacytoids DCs (pDCs), conventional DC (cDC)-restricted precursors named (pre-cDCs) or macrophages, through a common DC precursor (CDP) in the bone marrow that lost the capacity of generating monocytes. Throughout their life, DC traffic immense distances, they traverse a varied range of environments and barriers between different tissues and vessels to perform their function. Upon activation, DC precursors are challenged to leave the bone marrow niche, entering and seeding all tissues and migrating to lymphoid tissues to begin the adaptive immune response. Final differentiation into immature DCs is thought to occur after leaving the blood and seeding organ tissues (O’Doherty et al., 1994; Geissmann et al., 2003; Ginhoux et al., 2009; Liu et al., 2009). Extravasation of DC precursors involves coordinated signaling through cytokines, selectins and integrins (Springer 1994; Pendl et al., 2002; Alvarez et al., 2008) and it is most probably orchestrated by tetraspanins (Saiz et al., 2018; Yeung et al., 2018). In response to tethering, circulating (pre-)DCs expressing isoform L of selectin (L-selectin), and endothelial cells expressing E and P- selectin isoforms are required for leukocyte ‘rolling’ and homing of lymphoid and peripheral tissues (Tedder et al., 1995a), as suggested from analysis of L-selectin- or P-selectin-deficient mice (Mayadas et al., 1993; Arbonés et al., 1994). Tetraspanin CD63 is a key partner protein for P-selectin, as shown by reduced surface expression and clustering of P-selectin in CD63-deficient endothelial cells (Doyle et al., 2011). Later, DC precursor adhesion is achieved after binding of chemoattractants expressed by blood endothelium to CX3CR1 on pre-DCs (Geissmann et al., 2003; Auffray et al., 2007). This leads to activation of α4β1 and β2-Integrins by their ligands; ICAM-1/2, VCAM-1, and MAdCAM-1 that are expressed on blood endothelial cells (Springer 1994). With this, cells remain adhere and arrested, they leave the blood vessels thought-out diapedesis (Springer 1994).

It has been described that regulation of α4β1 and/or β2-Integrins is performed by five tetraspanin family isoforms; CD9, CD37, CD53, CD81, CD82, and CD151 on leukocyte (Shaw et al., 1995; Mannion et al., 1996; Shibagaki et al., 1999; Karamatic Crew et al., 2004; van Spriel et al., 2012; Reyes et al., 2015; Wee et al., 2015; Franz et al., 2016; de Winde et al., 2020; Dunlock 2020). Nevertheless, Cd81 and Cd37 -deficient mice display normal immune system development (Maecker and Levy 1997; Knobeloch et al., 2000), suggesting that compensation mechanisms through other tetraspanins can occur or that tetraspanins might not be required for DC precursor cells to seed peripheral tissues (de Winde et al., 2020).

### 3.2 DC phenotypes

#### 3.2.1 Immature DCs

By navigating tissues immature DCs function as sentinels, and warn the immune system of tissue damage or infection signs. They first scan peripheral tissues (so-called “patrolling” function), before migrating through the lymphatics to secondary lymphoid tissues (such as lymph nodes) (Germain 1994; Matzinger 2002; Schulz and Reis e Sousa 2002). Importantly, DCs can identify tumor antigens produced by cancer cells (Flamand et al., 1994; Fields et al., 1998), also playing a strategic role in initiating an anti-cancer immune response (Coulie et al., 2014). DC maturation is caused by uptake of foreign antigens, leading to migration to lymphoid tissues where they interact with and activate T cells to initiate adaptive immunity (Ingulli et al., 1997; Bousso and Robey 2003) through lymphatic vessels (Larsen et al., 1990; Granucci et al., 1999).

DCs are the best example of ameboid myeloid cells, and they are widely represented: immature DCs set habits in every tissue, performing receptor-mediated phagocytosis or non-specific macropinocytosis to sample ambient antigens (Reis e Sousa et al., 1993; Sallusto et al., 1995). This is the essence of their immune sentinel function but comes at the expense of limited migratory capacity (Banchereau and Steinman 1998). After DCs identify a possible danger, they stop macropinocytosis, and become highly migratory (For a review, see (Delgado et al., 2022)). DCs organize at the leading edge actin-rich protrusions that allow the cell to progress across the tissues, a process that occurs concomitantly with the so-called cell “flowing, or a trailing edge passive movement (Lämmermann et al., 2008). Passing through narrow spaces leads to cell “squeezing,” is allowed by the motor protein myosin II, leading to cell rear contraction, resulting in forward nucleus movement. DCs have been shown to migrate quickly in an amoeboid-like manner, exploiting actomyosin contractility *via* the cell cortex to modify their shape persistently (Lämmermann and Sixt 2009; Renkawitz et al., 2009). This motility fashion is clearly independent of integrins because it has been reported that integrin inactivation, *via* deletion of Talin and all integrin heterodimers, involved in, has no impact on DC migration *in vivo* or in three-dimensional spaces (Lämmermann et al., 2008).

Immature DCs, which are responsible for patrolling tissues, tend to move at variable speeds as they display an intermittent migration mode (Faure-André et al., 2008), due to the myosin IIA antagonistic effects exerted in antigen capture by macropinocytosis *versus* cell migration. Studies performed using microfluidic devices–in which is possible to separately alter myosin IIA activity at the front and back of the cell–showed that during phases of the slow movement, accumulation of myosin IIA was observed at DCs front and was recruited to the macropinosomes used for antigen capture. Disturbing within the cell the normal front-to-back myosin gradient, slows down DC migration as a result of myosin IIA enrichment in the cell front controlled by the MHC class II-associated protein invariant chain (CD74) (Chabaud et al., 2015). The myosin localization at the DC front is essential for antigen capture by macropinocytosis, as revealed by the less efficient formation and retrograde intracellular transport of antigen-loaded macropinosomes observed in both CD74-deficient and myosin II-deficient DCS (Chabaud et al., 2015). Recruitment of Myosin IIA to macropinosomes requires an Arp2/3-nucleated branched actin (Pollard and Borisy 2003; Vargas et al., 2016). Inhibition or knock-out of Arp2/3 complex in immature DCs leads to F-actin reduction at the cell front decreased antigen capture but enhanced migration (Vargas et al., 2016). Thus, the intermittent migration of immature DCs facilitates effective antigen internalization during space exploration by these cells.

#### 3.2.2 Mature DCs

Dendritic cells enter into a maturation program upon encounter with a dangerous signal. Upon maturation, several processes linked to the sentinel function are downregulated, amongst them is macropinocytosis. DC further increase the surface expression of many molecules upon maturation, most of them facilitating antigen presentation, cell migration, and chemotaxis to lymph nodes DC homing to lymph nodes at steady-state and upon inflammation is tightly dependent on the upregulation of the G-protein coupled chemokine receptor 7 (CCR7) (Förster et al., 1999; Ohl et al., 2004). Both CCL19 and CCL21 are ligands of CCR7, while CCL21 is critical for DC migration from peripheral tissues to lymphatic vessels and lymph nodes, CCL19 allows proper localization of DC in lymph nodes (Wendland et al., 2011). In peripheral tissues, endothelial cells secrete CCL21, which allows the formation of CCL21 haptotatic gradients in the direction of lymphatic vessels (Weber et al., 2013). Endothelial cells upregulate the expression of CCL21 upon inflammation, which increases the arrival of DC to the nearest lymph node (Johnson and Jackson 2010). CCL21 gradients start at approximately 90 μm from lymph vessels, which tails the distance that shifts DC migration from random to directional (Weber et al., 2013). CCL21 is additionally released at the site of transmigration and promotes the entry of mature DC into lymphatic vessels (Vaahtomeri et al., 2017). The CCL21 gradient within lymphatic vessels also facilitates the migration of mature DC to lymph nodes (Russo et al., 2016). The role of pro-inflammatory mediators in DC activation has been widely studied. For example, PGE2 induces CCR7 oligomerization on the DC surface, which leads to CCR7 phosphorylation by Src kinases and increases the efficiency of DC migration along CCL21 gradients (Hauser et al., 2016).

Remarkedly, to be able to efficiently follow these gradients, DCs must increase their cell-intrinsic migration speed and persistence (Vargas et al., 2016).

For mature DC to reach their final destination, lymph nodes, they first need to enter lymphatic vessels through intravasation. The first step in this process is to cross the vessel’s basement membrane. It has been shown that for this, DC search for entry points or portals along the discontinuous structure of the membrane. Once they have found a portal, DC send protrusions into the vessels and contracts their cell rear to squeeze and penetrate its lumen (Pflicke and Sixt 2009).

Thus, DC maturation induces major actin cytoskeleton rearrangements, which enhances DC motility to permit a fast migration (Vargas et al., 2016). In contrast to the homeostatic situation, in mature DCs most of the F-actin is located at the cell rear (Vargas et al., 2016) and it is critically maintained by the Formin protein family member mDia1, which is activated by the small GTPase RhoA, thereby ensuring fast migration. It has been shown that mDia1- deficient DCs display impaired chemotaxis of mature DCs along CCL21 gradients *via* lymphatic vessels to the lymph nodes (Vargas et al., 2016). Upon systemic inflammation, blood circulating DCs change their ameboid behavior. The integrin ligands expression (e.g. ICAM1 and VCAM1) is upregulated by the lymphatic endothelium hence promoting the adhesion-mediated DC transmigration (Johnson et al., 2006; Johnson and Jackson 2010; Vigl et al., 2011). Monoclonal antibodies based blocking of β2-Integrin, in presence of the pro-inflammatory cytokine TNF-α, leads to a reduction in DC transmigration (Johnson and Jackson 2010). The adhesion molecule L1 (also known as CD171 or L1CAM) is additionally expressed on the surface of some DC subsets, like in Langerhans cells (Pancook et al., 1997; Maddaluno et al., 2009) and *via* intercellular binding to integrins or L1, it is involved in neuronal cell movement and cell-cell adhesion (Maness and Schachner 2007). L1-deficient DCs adhere less to the endothelium and show defects in transmigration across the lymphatic endothelium (Maddaluno et al., 2009), suggesting that L1 has a role in blood DC intravasation.

Lymphatic endothelial cells (LECs) express high levels of the lymphatic vessel endothelial protein LYVE-1, a receptor for hyaluronic acid (HA), which is bound at the DC surface. This interaction between HA and LYVE-1 facilitates DC entry into the lymphatic capillaries (Johnson et al., 2017). Upon inflammation, LECs also upregulate the expression of integrin ligands such as ICAM-1, which promotes an adhesion-mediated DC transmigration (Johnson et al., 2006; Johnson and Jackson 2010; Vigl et al., 2011). Finally, the interaction between DC and LECs increases intracellular calcium signaling, leading to the secretion of vesicles loaded with CCL21, thereby reinforcing dendritic cell recruitment and entry into the lymphatics (Vaahtomeri et al., 2017). Once DC enter lymphatic vessels, which forms an incredibly complex network, they need 12–24 h to arrive at lymph nodes. Indeed, DC moves slowly within lymphatics along the vessel wall by extending protrusions at the cell’s front (Tal et al., 2011).

Once the maturation state is achieved, potentially, an increase in DCs macropinocytosis might be triggered in response to extracellular fluid increased volume, hence facilitating effective sentinel movement during inflammation (de Winde et al., 2020). Indeed, DC migration towards body tissues is an integrin-independent process and raises interesting questions regarding the migration mechanical forces, because when migrating cells exert large forces upon the surfaces, hence reducing their sensitivity to minor forces (Balaban et al., 2001; Legant et al., 2010; Moreau et al., 2019). Whereas, adhesion-independent migration exert significantly smaller forces on the substratum (Bergert et al., 2015; Moreau et al., 2019), are DC sensitive to the small forces? It has been shown that hydraulic resistance produced by fluid displacement as cells progress towards the tissue, coupled with geometric confinement, represents the major factors that restrict DC movement within tissues. Because immature DCs constitutive engulf the extracellular fluid non-specifically *via* macropinocytosis they have a decreased sensitivity to hydraulic resistance (Sallusto et al., 1995; Moreau et al., 2019). Macropinocytosis inhibition has been shown to restore barotaxis (i.e. cell’s tendency to move along the path of least hydrostatic resistance) in immature DCs fields (Moreau et al., 2019). Tissues with high hydraulic resistance that otherwise may be unreachable, can be patrol tissues exhaustively by this immature DCs special feature.

### 3.3 DCs subtypes

#### 3.3.1 Coventional DCs

cDCs constitute a small subset of tissue hematopoietic cells that reside in most lymphoid and non-lymphoid tissues, and are characterized by an enhanced capacity of sensing tissue injuries, phagocytosis of environmental and cell associated antigens, that later are presented to T lymphocytes. Thus, cDCs induce immunity to any tissue foreign antigens and generate tolerance to self-antigens. They can be divided into migratory DCs, which reside in non-lymphoid tissues and lymphoid tissue-resident DCs. They have two main functions: maintain tolerance to self and induce specific immune responses against antigens (Merad et al., 2013). Then, some of the key features of cDCs are; (1) their capacity of constantly capture tissue and blood antigens in non-lymphoid tissues and in the spleen marginal zone in homeostasis. The cDCs found in healthy non-lymphoid tissue, i.e. the skin, are not ‘resting’, instead they are permanently going into a maturation process known as ‘homeostatic migration’ that drives them into the draining lymph nodes (Wilson et al., 2008) at lower frequency (Shortman and Naik 2007). (2) Their unique dotation for processing and presenting antigens (Villadangos and Schnorrer 2007; Segura and Villadangos 2009; Joffre et al., 2012). Following homeostatic maturation, non-lymphoid tissue cDCs upregulate their expression of MHC class II molecules and they can transport cutaneous self-antigens to the T cell zones of the draining lymph node (Hemmi et al., 2001). The homeostasic migration event of non-lymphoid tissue cDCs leads to upregulation of MHC class II molecules as they can transport cutaneous self-antigens to the draining lymph nodes T cell zones (Hemmi et al., 2001) and is governed by the expression of the chemokine receptor 7 (CCR7) on their surface (Ohl et al., 2004). (3) A superior capacity to migrate, even when they are loaded with antigens, to a T cell zone in the lymph node during homeostasis and during inflammation (Förster et al., 2012). Once in the lymph node, they are capable of priming naive T cell responses (Banchereau and Steinman 1998). In most mouse peripheral tissues, including the skin and the gut, DCs constitute a network of trafficking from which they are able to enter or exit from non-lymphoid organs.

The skin is composed by two anatomically different layers: the epidermis, a tightly packed stratified epithelium formed by keratinocytes generating the water impermeable *stratum corneum*, and the dermis, formed by fibroblasts, collagen and elastin fibers. Both layers are separated by a basement membrane. In the epidermis, it is localized a unique population of DC, known as Langerhans cell (LCs), that expresses C-type lectin langerin (CD207) and represents the exclusive tissue-resident DC population in the epidermis. LCs are localized in the interfollicular epidermis and the epithelium of the hair follicles, which are dense in mouse, whereas in the human skin, larger areas of interfollicular skin sparse with hair follicles. In homeostasis the dermis is populated by several types of DCs, including; (1) XC-chemokine receptor 1 (XCR1)^+^ conventional DC1s (cDC1s) the only subset to express high levels of langerin in the dermis. Of note, the human dermis XCR1^+^ dermal cDCs co-express CD141 (also known as thrombomodulin and BDCA3) and XCR1 (Haniffa et al., 2012). (2) CD11b^+^ conventional DC2s (cDC2s), (3) XCR1^−^CD11b^−^ double negative cDCs (Malissen et al., 2014; Tan et al., 2015). Whereas, during inflammation, CCR2^+^ monocyte- derived DCs (moDCs) also expressing CD11b are recruited to the dermis.

In the intestine, DC migration contributes importantly to tissue-homeostasis and immune surveillance of the gut, maintains tolerance towards proteins form food and commensal organisms. Within the small intestinal mucosa are located the lamina propria, Peyer’s patches and solitary intestinal lymphoid tissues, from each of them migratory intestinal DCs are derived. The largest compartment is generated by the small intestine lamina propria DCs, and that is composed (at least) by three different cDCs populations, all of them are migratory and reside in the intestine derived lymph (Cerovic et al., 2013) and the intestine-draining mesenteric lymph nodes. It has been shown that homeostatic migration from the lamina propria-derived towards the mesenteric lymph nodes is dependent of CCR7 signaling (Pabst et al., 2006; Schulz et al., 2009) and is significantly amplified in response to TLR triggering and inflammatory cytokine (Yrlid et al., 2006). Additionally, in the lamina propria reside the intestinal cDC1s CD103^+^CD11b^−^ and intestinal cDC2s CD103^+^CD11b^+^ subpopulations (Persson et al., 2013). Both lamina propria-derived CD103^+^ cDCs have been shown to induce response in regulatory T cells, and this induction is depend on retinoic acid (Coombes et al., 2007; Sun et al., 2007). CD103^+^ CD11b^−^ DCs share features with mouse splenic CD8^+^ DCs: they have a remarkably efficiency at cross-presenting antigens to activate CD8^+^ T cells, and they express Clec9, a molecule that recognizes F-actin released from necrotic cells, and TLR3, which binds double-stranded RNA. These cells are also able to cross-present skin-associated self-antigens to CD8^+^ T-cells, suggesting a potential role in tolerance to self under homeostatic conditions (Heath and Carbone 2013). Whereas the CD103^+^ CD11b^−^ DCs population was shown to be crucial during early phase of infection with *Salmonella enterica* serovar Typhimurium when bacterias are disseminating to initiate a systemic infection (Erazo et al., 2021). This population is also the main producer of CCL17 cytokine which induces DC migration from the skin, amplifying their responsiveness to CCR7 and its ligand (Stutte et al., 2010). Also, it has been shown that upon skin infection with herpes simplex virus (HSV), CD103^+^CD11b^−^ DCs present antigens preferentially to CD4^+^ T-cells (Mount et al., 2008). Finally, two small subsets have been described; CD103^−^CD11b^+^CX3CR1^mid^ cDCs that reside in the lamina propria small intestine of mouse and human (Cerovic et al., 2013; Scott et al., 2015) and a ‘double negative’ CD103^−^CD11b^−^ DCs that has been shown to be present in the intestinal lymph (Cerovic et al., 2013).

DC-mediated transport of antigen from the intestine leads to T cell priming in mesenteric lymph nodes after oral antigen administration (Johansson-Lindbom et al., 2005; Worbs et al., 2006). It has been shown that upon challenge with luminal *Salmonella* bacteria, CD103^+^CD11b^+^ intestinal DCs are the first to acquire bacterial antigen and are recruited from the lamina propria into the intraepithelial cell layer after active migration (Farache et al., 2013). Recently, it has been shown that lamina propria CD103^+^CD11b^+^ cDC2s are imprinted by ‘environmental cues’ according to their position within the tissue, these cues include food-derived retinoic acid (ATRA) and the mucus component Muc2. Lamina propria cDCs display a mature-like proinflammatory phenotype; promoting T cell activation, whereas intraepithelial CD103^+^CD11b^+^ cDC2s exhibited an immature-like phenotype as well as tolerogenic properties as they trigger T cell anergy (Rivera et al., 2022). These different phenotypes raised from the action of ATRA, which improved actomyosin contractility and stimulated lamina propria resident cDC2 transmigration into the epithelium. This implies that by reaching distinct niches inside the tissue, DCs can exist as immature and mature cells within the same tissue, constituting an efficient diversification mechanism within the tissues.

Lymphoid-resident DCs present antigens in lymphoid organs; the thymus, lymph nodes, the spleen, and in Peyer’s patches. They are usually classified into CD8^+^cDCs and CD8^−^ cDCs, also classified into CD4^+^CD8^−^ (CD4^+^) and CD4^−^CD8^−^ cDCs. In lymphoid organs and in addition to lymphoid resident cDCs, two subsets of migratory DCs have been characterized in mice: Integrin αE/CD103^+^ and Integrin αM/CD11b^+^ (Shortman and Naik 2007). Typically, lymphoid-resident DCs display an immature phenotype with high antigen uptake capacity whereas migratory DCs appear to have mature-like phenotype once they reach lymph nodes (Wilson et al., 2003).

#### 3.3.2 Langerhans cells

(LCs) are the DCs that reside on the stratified epithelia (Helft et al., 2010), which are concentrated in the epidermis where they co-exist in intimate association with the keratinocytes, the main epidermal cell type. LCs are also found in other stratified epithelia, such as the genital epithelium and oral cavity mucosa, but they rise from a different precursor and are functionally different (Capucha et al., 2015). In homeostasis, they continuously extend membrane protrusions to patrol their environment, and upon encountering a pathogen, they engulf it using these large and dynamic dendrites (Ng et al., 2008). Epidermal Langerhans cells express high levels of langerin, which has been shown to have a key role in the uptake of several pathogens including type I of human immunodeficiency virus (HIV1) (Turville et al., 2002). LCs are also characterized by the expression of E-cadherin, EpCAM, DEC205 and the presence of “mysterious” granules called Birbeck granules whose exact function remains to be discovered. In homeostasis, LCs constantly migrate to lymph nodes with a rate that increases upon inflammation (Heuzé et al., 2013).

LCs were originally described according to functional criteria as a part of the DC compartment, but today they are considered to be derived from embryonic precursors as a subset of tissue-resident macrophages (Hoeffel et al., 2012; Doebel et al., 2017). The precise LCs function during an immune response has not been fully clarified despite quick progress in the field. Using developmental origin as a distinguishing feature, LCs can defensibly be classified in the macrophage lineage (Guilliams et al., 2014). Even though ontogenic similarities are shared with tissue-resident macrophages, LCs unlike macrophages, constantly migrate to draining lymph nodes to present self-antigens and establish immune tolerance in homeostatic conditions (Ghigo et al., 2013; Gosselin et al., 2014), but as tissue-resident macrophages; they retain a unique self-renewal capacity within the epidermis (Cumberbatch et al., 2001).

In homeostasis, LCs reside within epithelial layers and establish one of the initial lines of immunological defense against pathogens (Deckers et al., 2018). Without threatening, LCs change continuously shape by extending and retracting protrusions within the intercellular spaces and also between epidermal cells, establishing a dense network across the epidermis. This dynamic behavior allows large epidermis sampling at the same time as the remaining sessile (Kissenpfennig et al., 2005; Nishibu et al., 2006). Both in steady-state conditions and during inflammation LCs can take up and process foreign antigens, which in the lymph nodes are presented to cells triggering the adaptative immune response.

When LCs are challenged, they undergo a series of changes that allows them to migrate. Initially, they weaken their intercellular association with the surrounding keratinocytes through the release of E-cadherin, yielding to β-catenin translocation, which is involved in the tolerogenic phenotype of DCs (Schuler and Steinman 1985). Also, as LCs interact with ECM components of the dermis and lymph nodes. It was shown that α-Integrins regulate the initial stages of LC migration out of the epidermis across the underlying basal membrane (Price et al., 1997). This implies that LCs behave differently from DC ameboid sentinels, because they require integrins on the LC surface to regulate LC migration from the epidermis towards the draining lymph nodes. It seems that, similarly to macrophages in the epidermis. LCs locally secrete collagen-degrading matrix metalloproteinases, facilitating reorganizations to reach the dermis after crossing the underlying basement membrane, enter the afferent lymphatics to enter into the T cell area in the draining lymph node. In so doing, LCs (Nagao et al., 2012; Deckers et al., 2018)

Nagao et al. showed that an intermediate population of LC-precursor is recruited *via* hair follicles to the epidermis for replenishment upon LC migration to lymph nodes. The authors analyzed the repopulation of the skin by LCs in transgenic mice conditionally depleted of langerin-expressing cells (langerin being a Langerhans cell marker). As previously described, in absence of threat, this is a physiological process, but it is accelerated by treating the skin with a hapten to induce inflammation.

After LCs depletion, external stress (induced by stripping the cornified skin sheets with adhesive tape) rapidly induced the accumulation of leukocytes near hair follicles. At later time points, LCs could be seen to extend dendrites or migrate into the inter-follicular epidermis (Woodman 2012). Interestingly, another myeloid cell population was found in higher numbers at the epidermis following LC depletion, featured by expression of GR1 as opposed to langerin, EPCAM (another LC marker), and MHC class II molecules, and was suggested to be an immediate precursor of an LC sub-population (which are GR1^-^) and were originated from bone marrow-derived GR1^hi^ monocytes. Using bone marrow chimeras, it was shown that these cells were different from CX3C-chemokine receptor 1 (CX3CR1)-expressing LC precursors that populate the skin during embryonic development. Also, this LCs reseeding process from the bone marrow into the epidermis elapses across precisely defined paths along the hair follicle (Nagao et al., 2012). Maturation of LCs, is achieved by an increased expression of actin concomitantly with additional actin filaments formation to allow the formation of dendritic structures and migration of formerly sessile cells, (Ross et al., 1999).

#### 3.3.3 Plasmacytoid DCs

(pDCs) express relatively low levels of MHC class II and co-stimulatory molecules (Heuzé et al., 2013). In homeostasis. pDCs are absent from the skin and they are only recruited in inflamed skin where they promote wound repair (Gregorio et al., 2010) and also participate in the systemic pro-inflammatory response triggered after stimulation with Toll-like receptor 7 (TLR7) agonists (Guiducci et al., 2010). Then, pDCs they accumulate principally in lymphoid organs and whose major function is, in response to viral infections, to secrete high amounts of interferon-α (IFN-α), prior to differentiating into mature DCs that are able to prime T cells against viral antigens (Liu 2005; Merad et al., 2013; Mildner and Jung 2014; Schlitzer and Ginhoux 2014; Sander et al., 2017). pDC migration has been shown different from that of cDCs. Additionally, to secondary lymphoid organs, pDCs can also migrate from blood into peripheral tissues. pDC migration depend CD62L, PSGL1, β1 and β2 integrins and multiple chemokine receptors, such as CXCR4, CCR7, CXCR3, CCR5, CCR2, CCR6, CCR10 and CCR9 (Sozzani et al., 2010; Seth et al., 2011). Also, another difference is that pDCs derived from BM traffic into the blood and circulate through high endothelial venules, instead of afferent lymphatics as cDCs, until reaching T cell areas inside the lymph nodes (Penna et al., 2001; Sozzani et al., 2010).

#### 3.3.4 Monocyte-derived dendritic cells

(MoDCs), are the DCs that differentiate from monocytes upon inflammation (Geissmann et al., 2010). Monocytes constitute about 10% of leukocytes in human blood and 4% of leukocytes in mouse blood (Guilliams et al., 2018). Upon certain infections, the number of circulating monocytes increase and they infiltrate into the infected tissue or organ (Qu et al., 2014), where they differentiate into DCs (Schlitzer et al., 2015). There are four general subsets of monocytes according to their surface expression of CD14 and CD16: CD14^high^ CD16^−^, CD14^high^ CD16^low^, CD14^low^ CD16 ^high^, and CD14^low^ CD16^low^ (Ziegler-Heitbrock et al., 2010).

Human MoDCs, as well as the previously described subpopulations of DCs, have a migratory capacity toward the lymph node dependent on surface CCR7 expression in response to cytokines CCL19 and CCL21, but additionally requires prostaglandin E2 (PGE2) (Scandella et al., 2002). Proinflammatory mediator PGE2 has been shown to be crucial for MoDCs to acquire substantial chemotactic responsiveness to lymph node–derived chemokines and develop potent T-helper cell stimulatory capacity (Scandella et al., 2002).

## 4 Tissue-resident macrophages

### 4.1 Brain microglia

Microglia are central nervous system resident macrophages. Under physiological conditions, microglia are in a state known as “ramified’ and present processes that are being dynamically extended and retracted. They change to an “amoeboid” status if there is a neuronal injury, retracting their protrusions; this state includes migration and accumulation of microglia at the site of damage (Okajima and Tsuruta 2018). Additionally, some microglia display “intermediate” status, mixing functions between “ramified” and “amoeboid.” In the ramified form, microglia without translocating their cell body move their processes, whereas, in the amoeboid form, the entire microglia is able to migrate towards the brain tissue (Kettenmann and Verkhratsky 2011). Two-photon brain imaging and single-cell RNA sequencing have revealed that heterogeneity of microglia status, defined by their morphology and functionality, depends on the brain region in an age-dependent manner. However, the relation between microglial morphology and function and the molecular mechanism involved remains controversial (Okajima and Tsuruta 2018), whether these migration fashions are distinctly regulated during development and in response to pathology remains to be elucidated. Several candidates to represent a signal for microglia during pathological events occurring in the brain have been suggested, including the chemokine CCL21 (Rappert et al., 2002), ATP (Honda et al., 2001; Davalos et al., 2005), morphine (Takayama and Ueda 2005), lysophosphatidic acid (Schilling et al., 2004), cannabinoids (Walter et al., 2003), and bradykinin (Ifuku et al., 2007). Also, transporters and ion channels related to the actin cytoskeleton during cell migration have crucial roles (i.e K^+^, Cl^−^ channels, Na^+^/Ca^2+^, Na^+^/H^+^, Cl^−^/HCO3^−^ exchangers, and Na^+^/HCO3^−^ co-transporter and Ca^2+^-permeable stretch-activated cation channels) and whose role in microglial migration remains to be analyzed in detail (Schwab 2001a, b; Ifuku et al., 2007)

Real-time imaging based on two-photon laser microscopy has revealed that microglial processes, highly dynamic, move randomly, and quickly towards the brain parenchyma (Davalos et al., 2005; Nimmerjahn et al., 2005). Local brain ATP injection has been used to analyze the chemotaxis of microglial processes, that rapidly orient to the site of damage. This behavior can be reproduced by performing a laser ablation and can be stopped by the ATP-hydrolyzing enzyme apyrase or by G protein-coupled purinergic receptors and connexin channels blockers, that are highly expressed in astrocytes, suggesting ATP as primarily stimulator of astrocytes, and later of microglial cells (Davalos et al., 2005).

Pathological conditions (i.e., stroke, lesion, tumor invasion, or neurodegenerative disorders), trigger microglial activation and migration to lesions. Studies in isolated living slices from adult brain-injured mice, showed 24 h after injury the widespread signs of perilesional microglia migration, which peaked at 3 days with ∼5 μm/min average migration speed and maximum speed of >10 μm/min., similar to the average speed of an immature DCs (5–8 μm/min) (Maiuri et al., 2012), but it was mostly achieved in a random-walk migration fashion, in opposition to the directional chemoattract-guided migration expected (Carbonell et al., 2005a, b).

Furthermore, microglia’s closely related features to macrophages as crucial mediators of the immune response in the brain, because they are subject to suffering striking functional and morphological changes after central nervous system threat. Microglia express many neurotransmitter receptors and could be stimulated by excitatory neurotransmitters. It has been described that cultured microglia stimulated with glutamate receptor agonist kainate (KA) experienced dramatic cytoskeletal rearrangements; cytoplasmic redistribution of actin filaments and bundles, as revealed by phalloidin staining. This suggests changes in migration and phagocytosis of microglia cells trigger by a glutamate receptor agonist (Christensen et al., 2006).

On the other hand, it has been also shown that cultured human microglia expressed receptors for the complement fragments C3a and C5a (Lacy et al., 1995), known anaphylatoxins able to trigger the complement system activation, an important event during and immune response (Noris and Remuzzi 2013). C5a receptor expression can be greatly upregulated in the spinal cord microglia following peripheral nerve injury or in a brain undergoing inflammation (Griffin et al., 2007), whereas in a healthy brain remains marginally expressed (Gasque et al., 1997). This C5a receptor has been proposed as a regulator of microglial migration, because microglial cells in murine culture, after treatment with C5a quickly (within seconds) trigger membrane ruffling together with rearrangement of the actin cytoskeleton that allows lamellipodia extension independent from the concentration of Ca^2+^ intracellular (Nolte et al., 1996). These phenomena can be inhibited by cytochalasin B suggesting an important role for the cytoskeleton (Nolte et al., 1996).

Additionally, it has been shown that stimulation of CXCL12 / CXCR4 pathway can trigger microglial migration and is accelerated by brain hypoxia, due to increased CXCR4 expression induced by the ‘hypoxia inducible factor-1α’ (HIF-1α) activation and PI3K/Akt signaling pathway (Wang et al., 2008). Following a hypoxic threat in neonatal rats, amoeboid microglial cells increase the production of monocyte chemoattractant protein-1 (MCP-1) through the NF-κB signaling pathway, leading to migration (from the surrounding areas) and amoeboid microglial cells accumulation into the periventricular white matter (Deng et al., 2009).

### 4.2 Liver Kupffer cells

Kupffer cells are commonly thought sessile tissue of the liver and constitute the greatest pool of resident macrophages in the body, accounting for around 30% of nonparenchymal liver cells, playing an crucial role in phagocyting foreign substances contained in the systemic circulation (Brouwer et al., 1995).

The role of Kupffer cells acting as antigen-presenting cells by engulfing external particles, was shown *in vivo* by a high-resolution video microscopy (MacPhee et al., 1992). They were shown to be able to independently migrate, in directions different from those of surrounding Kupffer cells, along sinusoidal walls, with a mean speed of migration of 4.6 ± 2.6 (SD) μm/min (*n* = 29 migrating Kupffer cells) that remain constant when migration assay was performed following or not the flow. The phagocytic challenge study *in vivo* was done by presenting fluorescent microspheres and showed that Kupffer cells that engulfed a few microspheres migrated slowly (∼1 μm/min, *n* = 10), compared with cells that engulfed many microspheres, which were unable to migrate (MacPhee et al., 1992).

Interestingly, Kupffer cell functions impaired by aging are thought to be responsible for the susceptibility to sepsis followed by infection, trauma, or tumorigenesis observed in old people. A study shows that the distribution and contents of cytoskeleton-forming proteins are diminished by age in Kupffer cells. In 24-month-old Kupffer cells actin, myosin and vimentin were significantly decreased to 68%, 85%, and 76% respectively, compared with freshly derived Kupffer cells. Furthermore, a quantitative evaluation of polystyrene beads phagocytosis by primary cultured Kupffer cells at several ages evidenced significant decreases in phagocytic activity as cells age (Sun et al., 1998). Whether their migration is impaired remains to be elucidated.

### 4.3 Lung alveolar macrophages

Alveolar macrophages (AMs) establish the primary line of phagocytic defense in the alveolus (lower airways), and are continuously exposed to inhaled substances or pathogens as a result of their exposed position in the alveolar lumen (Féréol et al., 2008). AMs are large mononuclear, powerful phagocytic cells found on the alveolar surface. Similar to other leukocytes AMs are mobile and easily recruitable immune cells that fight aggressively against infection. Nevertheless, they are also present in absence of infection or particulate threat. Despite their position as the primary antigen-exposed immune cell population, and their range of immunoregulatory functions, AMs were not commonly thought to contribute to adaptive immune responses. It was widely believed that AMs were unable to migrate from the alveolar spaces to lung draining lymph nodes. Instead, constitutive CCR7-dependent migration of pulmonary DC was thought to be the only mechanism by which particulate antigen is transported from the lungs draining lymph nodes (Legge and Braciale 2003; Jakubzick et al., 2006). Nevertheless, their role in the adaptive immunity to inhaled material, including pathogens, was shown in lung draining lymph nodes following transport of antigen to this site (Kirby et al., 2009).

Assessment of the F-actin cytoskeleton, (revealed by intensity of phalloidin staining), revealed AM population heterogeneity and described four types of AM phenotypes (A-D) according to SP-A genotype, sex, and age , and degree of AM exposure to SP-A1 or SP-A2 (acutely or chronically) (Tsotakos et al., 2016). Since for the moment, there is no direct evidence supporting a functional significance of these sub-populations, it is possible that they represent AM different activation stages. The globular actin (G-actin) -based AM intensity and measurements changed among the F-actin-based AM subpopulations, suggesting that changes in cell phenotype can reflect alter gene expression instead of only cytoskeleton rearrangements (Tsotakos et al., 2016).

Pulmonary surfactant protein (SP), is a lipoprotein complex is essential for life, due to its capacity of diminishing surface tension at the alveolus air-liquid interface, preventing their collapse at lung low air volumes. SP is formed by a combination of phospholipids, non-serum-derived proteins, SP-A, SP-B, and SP-C (reviewed in (Floros et al., 2021). It has been described that surfactant protein A (SP-A) and its isoforms, can induce actin cytoskeleton modifications in AMs after microorganisms threat in the lung, contributing to immunity by regulating inflammation (Tino and Wright 1999). The pattern of F-actin fluorescence distribution (revealed by AlexaFluor phalloidin staining) is reduced in SP-A2 compared to SP-A1 (both human isoforms) and the effect on actin-related/cytoskeletal proteins is specific as shown *via* proteomic analysis (Phelps et al., 2013), suggesting differences in actin cytoskeletal processes. Specifically, in this study, the authors show that the ARP3—part of Arp2/3 actin nucleation complex that nucleates branched actin–is also diminished in SP-A2 AM, in agreement with lower F-actin levels (Floros 2021).

In the present COVID pandemic, AMs are in the limelight. It has been shown that SARS-CoV-2 infects AMs, which react by secreting T cell chemoattractant molecules (Grant et al., 2021). Recruited T cells induce inflammatory cytokine release from AM when producing Interferon-γ, a signal pathway leading to enhanced T cell activation. According to the latest models, during SARS-CoV-2 infection, AMs- containing SARS-CoV-2 and T cells establish positive feedback that persists together with an alveolitis (Grant et al., 2021).

## 5 Perspectives

Myeloid cells constitute an immune system main cellular compartment that comprises monocytes, DCs, tissue-resident macrophages, and granulocytes. In this review we have focused on how DCs, tissue-resident macrophages and their precursors use migration to fulfil their sentinel function. Indeed, cell migration is a fundamental feature to these cells, from their embryonic development to tissue homeostasis, immune surveillance, and wound healing (Yamada and Sixt 2019). Moreover, their aberrant migration can contribute to pathologies such as chronic inflammation (Qu et al., 2019), vascular diseases (Yu et al., 2018), and tumor development or growth (van Helvert et al., 2018).

We anticipate that future studies should shed light on the molecular mechanisms that control the migration of DC and macrophage precursors. Indeed, knowledge is limited there, most likely as a consequence of the challenges involved in obtaining enough cells and recapitulate the microenvironment in which they evolve in *in vitro*. Emergence of micro-fabricated tools that allow reconstitution of idealized migration conditions should help circumvent this problem (Charras and Sahai 2014). The use of these tools can further help comparing the requirements for migration of these different cell types in a “normalized” environment. The ‘world cell race’ (Maiuri et al., 2012) is an ‘iconic’ illustration, in which migratory conditions were standardized allowing comparison of migration speed and migration persistence on a one-dimensional (1D) ECM-coated surface of 50 cell types, leading to a universal (1D, 2D and 3D) comprehensive understanding (Maiuri et al., 2015). In addition, recent studies have revealed that cell confinement, strongly influence the migration of immune sentinels (Charras and Sahai 2014; Stroka and Konstantopoulos 2014; Hung et al., 2016). As confinement growths, cell deformation in order to squeeze into narrow spaces becomes more difficult. The nucleus, the stiffest cellular organelle, during confined migration has a rate-limiting role, preventing cell movement in ECM pores underneath a cell life-threatening threshold (Beadle et al., 2008; Wolf et al., 2013; Davidson et al., 2014; Harada et al., 2014). It has been shown that confinement causes nuclear deformation because it imposes a mechanical stress on it (Page-McCaw et al., 2007; Friedl and Wolf 2009), leading to localized nuclear envelope rupture and consequent DNA-damage, which is particularly relevant for tumor cells (Denais et al., 2016) and in DCs (Raab et al., 2016; Alraies et al., 2022). How the deformation incidents that immune sentinels experience during their life-time impact on their immune-surveillance function in the context of infection or cancer remains as an opened fascinating question to be addressed.
